# {4-Bromo-2-[(2-morpholinoeth­yl)imino­meth­yl]phenolato}iodido(methanol)zinc(II)

**DOI:** 10.1107/S1600536809009234

**Published:** 2009-03-19

**Authors:** Cheng-Li Han

**Affiliations:** a College of Chemistry and Chemical Engineering, Qiqihar University, Qiqihar 161006, People’s Republic of China

## Abstract

The title compound, [Zn(C_13_H_16_BrN_2_O_2_)I(CH_3_OH)], is a new mononuclear zinc(II) complex synthesized by the reaction of equimolar quanti­ties of 5-bromo­salicylaldehyde, 2-morpholinoethyl­amine and ZnI_2_ in methanol. The Zn atom is four-coordinate in a distorted tetra­hedral geometry, binding to a phenolate O and an imine N atom of the Schiff base ligand, the O atom of a methanol mol­ecule and one I^−^ anion. In the crystal structure, adjacent mol­ecules are linked through inter­molecular O—H⋯O hydrogen bonds, forming centrosymmetric dimers.

## Related literature

For the structures of related zinc(II) complexes, see: Ali *et al.* (2008 ▶); You (2005 ▶); Zhu & Yang (2008 ▶).
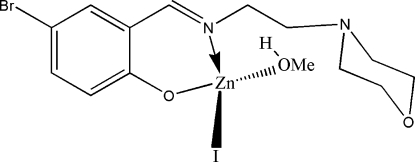


## Experimental

### 

#### Crystal data

[Zn(C_13_H_16_BrN_2_O_2_)I(CH_4_O)]
*M* *_r_* = 536.50
Monoclinic, 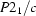

*a* = 7.747 (2) Å
*b* = 24.977 (3) Å
*c* = 9.598 (2) Å
β = 100.497 (4)°
*V* = 1826.1 (6) Å^3^
*Z* = 4
Mo *K*α radiation
μ = 5.24 mm^−1^
*T* = 298 K
0.30 × 0.30 × 0.28 mm

#### Data collection

Bruker SMART CCD area-detector diffractometer
Absorption correction: multi-scan (*SADABS*; Sheldrick, 1996 ▶) *T*_min_ = 0.217, *T*_max_ = 0.231
12877 measured reflections
3928 independent reflections
2994 reflections with *I* > 2σ(*I*)
*R*_int_ = 0.038

#### Refinement

*R*[*F* ^2^ > 2σ(*F* ^2^)] = 0.040
*wR*(*F* ^2^) = 0.096
*S* = 1.03
3928 reflections
203 parameters
1 restraint
H atoms treated by a mixture of independent and constrained refinement
Δρ_max_ = 0.96 e Å^−3^
Δρ_min_ = −0.71 e Å^−3^

### 

Data collection: *SMART* (Bruker, 1998 ▶); cell refinement: *SAINT* (Bruker, 1998 ▶); data reduction: *SAINT*; program(s) used to solve structure: *SHELXS97* (Sheldrick, 2008 ▶); program(s) used to refine structure: *SHELXL97* (Sheldrick, 2008 ▶); molecular graphics: *SHELXTL* (Sheldrick, 2008 ▶); software used to prepare material for publication: *SHELXTL*.

## Supplementary Material

Crystal structure: contains datablocks global, I. DOI: 10.1107/S1600536809009234/sj2595sup1.cif

Structure factors: contains datablocks I. DOI: 10.1107/S1600536809009234/sj2595Isup2.hkl

Additional supplementary materials:  crystallographic information; 3D view; checkCIF report

## Figures and Tables

**Table:** 

Zn1—N1	2.014 (3)
Zn1—O3	2.023 (3)
Zn1—O1	2.078 (3)
Zn1—I1	2.5346 (9)

**Table:** 

N1—Zn1—O3	114.78 (13)
N1—Zn1—O1	90.15 (12)
O3—Zn1—O1	90.42 (13)
N1—Zn1—I1	130.76 (10)
O3—Zn1—I1	113.36 (9)
O1—Zn1—I1	99.31 (9)

**Table 2:** Hydrogen-bond geometry (Å, °)

*D*—H⋯*A*	*D*—H	H⋯*A*	*D*⋯*A*	*D*—H⋯*A*
O3—H3*A*⋯O1^i^	0.84 (5)	1.81 (5)	2.649 (4)	178 (7)

Symmetry code: (i) 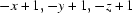.

**Fig. 1.**
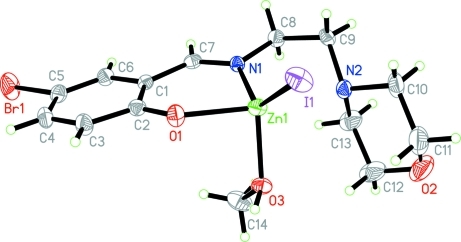
The structure of the complex, showing 30% probability displacement ellipsoids and the atom-numbering scheme.

**Fig. 2.**
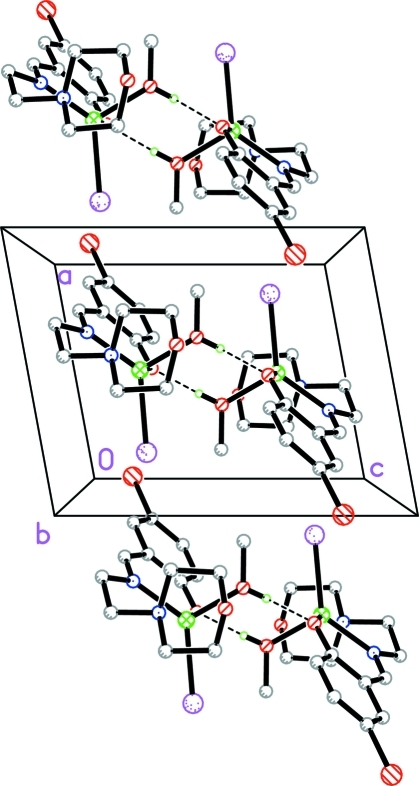
The crystal packing of (I) showing the formation of centrosymmetric dimers. Hydrogen bonds are shown as dashed lines.

## supplementary crystallographic information

### Comment

Metal complexes of the Schiff base 4-bromo-2-[(2-morpholinoethylimino)methyl]phenol have not been reported previously. In this paper, the author reports the crystal structure of the title compound, a new mononuclear zinc(II) complex, (I), Fig. 1.

In (I), the Zn atom is four-coordinate in a tetrahedral geometry, with one O and one imine N atoms of a Schiff base ligand, one O atom of a methanol molecule, and one I atom. The tetrahedral geometry is severely distorted, as evidenced by the coordinate bond lengths and angles (Table 1). The bond lengths and angles in this complex are comparable with those in the similar zinc(II) complexes (Ali *et al.*, 2008; You, 2005; Zhu & Yang, 2008). In the crystal structure, adjacent molecules are linked through intermolecular O–H···O hydrogen bonds (Table 2), forming centrosymmetric dimers (Fig. 2).

### Experimental

Equimolar quantities (1.0 mmol each) of 5-bromosalicyaldehyde, 2-morpholinoethylamine, and ZnI_2_ were mixed in methanol. The mixture was stirred at reflux for 30 min and filtered. The filtrate was slowly evaporated for a few days, yielding yellow block-like crystals.

### Refinement

H3A was located from a difference Fourier map and refined isotropically, with the O–H distance restrained to 0.85 (1) Å, and with *U*_iso_(H) values fixed at 0.08 Å^2^. The other H atoms were placed in idealized positions and constrained to ride on their parent atoms with C–H distances of 0.93–0.97 Å, and with *U*_iso_(H) set at 1.2 or 1.5*U*_eq_(C).

### Crystal data

**Table:**

[Zn(C_13_H_16_BrN_2_O_2_)I(CH_4_O)]	*F*(000) = 1040
*M**_r_* = 536.50	*D*_x_ = 1.951 Mg m^−^^3^
Monoclinic, *P*2_1_/*c*	Mo *K*α radiation, λ = 0.71073 Å
Hall symbol: -P 2ybc	Cell parameters from 3128 reflections
*a* = 7.747 (2) Å	θ = 2.6–25.8°
*b* = 24.977 (3) Å	µ = 5.24 mm^−^^1^
*c* = 9.598 (2) Å	*T* = 298 K
β = 100.497 (4)°	Block, yellow
*V* = 1826.1 (6) Å^3^	0.30 × 0.30 × 0.28 mm
*Z* = 4	

### Data collection

**Table:**

Bruker SMART CCD area-detector diffractometer	3928 independent reflections
Radiation source: fine-focus sealed tube	2994 reflections with *I* > 2σ(*I*)
graphite	*R*_int_ = 0.038
ω scans	θ_max_ = 27.0°, θ_min_ = 1.6°
Absorption correction: multi-scan (*SADABS*; Sheldrick, 1996)	*h* = −9→9
*T*_min_ = 0.217, *T*_max_ = 0.231	*k* = −30→31
12877 measured reflections	*l* = −12→12

### Refinement

**Table:**

Refinement on *F*^2^	Primary atom site location: structure-invariant direct methods
Least-squares matrix: full	Secondary atom site location: difference Fourier map
*R*[*F*^2^ > 2σ(*F*^2^)] = 0.040	Hydrogen site location: inferred from neighbouring sites
*wR*(*F*^2^) = 0.096	H atoms treated by a mixture of independent and constrained refinement
*S* = 1.03	*w* = 1/[σ^2^(*F*_o_^2^) + (0.0385*P*)^2^ + 2.0556*P*] where *P* = (*F*_o_^2^ + 2*F*_c_^2^)/3
3928 reflections	(Δ/σ)_max_ < 0.001
203 parameters	Δρ_max_ = 0.96 e Å^−^^3^
1 restraint	Δρ_min_ = −0.71 e Å^−^^3^

### Special details

**Table:**

Geometry. All e.s.d.'s (except the e.s.d. in the dihedral angle between two l.s. planes) are estimated using the full covariance matrix. The cell e.s.d.'s are taken into account individually in the estimation of e.s.d.'s in distances, angles and torsion angles; correlations between e.s.d.'s in cell parameters are only used when they are defined by crystal symmetry. An approximate (isotropic) treatment of cell e.s.d.'s is used for estimating e.s.d.'s involving l.s. planes.
Refinement. Refinement of *F*^2^ against ALL reflections. The weighted *R*-factor *wR* and goodness of fit *S* are based on *F*^2^, conventional *R*-factors *R* are based on *F*, with *F* set to zero for negative *F*^2^. The threshold expression of *F*^2^ > σ(*F*^2^) is used only for calculating *R*-factors(gt) *etc*. and is not relevant to the choice of reflections for refinement. *R*-factors based on *F*^2^ are statistically about twice as large as those based on *F*, and *R*- factors based on ALL data will be even larger.

### Fractional atomic coordinates and isotropic or equivalent isotropic displacement parameters (Å^2^)

**Table:**

	*x*	*y*	*z*	*U*_iso_*/*U*_eq_	
Zn1	0.50584 (7)	0.55343 (2)	0.28004 (5)	0.03432 (14)	
I1	0.17781 (4)	0.568293 (16)	0.25354 (4)	0.05466 (14)	
Br1	1.05025 (10)	0.31501 (2)	0.17245 (8)	0.0752 (2)	
O1	0.5139 (4)	0.47117 (12)	0.3133 (3)	0.0403 (8)	
O2	0.6023 (12)	0.73465 (19)	0.4160 (5)	0.119 (3)	
O3	0.6375 (4)	0.56560 (12)	0.4796 (3)	0.0370 (7)	
N1	0.6591 (4)	0.54528 (13)	0.1329 (4)	0.0276 (7)	
N2	0.5630 (5)	0.65133 (14)	0.2055 (4)	0.0381 (9)	
C1	0.7471 (6)	0.45205 (16)	0.1873 (4)	0.0298 (9)	
C2	0.6310 (6)	0.43807 (17)	0.2793 (5)	0.0340 (10)	
C3	0.6426 (7)	0.38546 (18)	0.3320 (5)	0.0464 (13)	
H3	0.5649	0.3747	0.3899	0.056*	
C4	0.7640 (8)	0.34916 (19)	0.3017 (5)	0.0502 (13)	
H4	0.7691	0.3148	0.3396	0.060*	
C5	0.8782 (7)	0.36454 (18)	0.2139 (5)	0.0409 (11)	
C6	0.8706 (6)	0.41432 (18)	0.1569 (5)	0.0364 (10)	
H6	0.9477	0.4237	0.0972	0.044*	
C7	0.7474 (6)	0.50294 (17)	0.1163 (4)	0.0310 (9)	
H7	0.8209	0.5054	0.0501	0.037*	
C8	0.6873 (6)	0.59135 (17)	0.0431 (5)	0.0366 (10)	
H8A	0.6722	0.5799	−0.0549	0.044*	
H8B	0.8066	0.6044	0.0713	0.044*	
C9	0.5603 (6)	0.63595 (17)	0.0561 (4)	0.0346 (10)	
H9A	0.5897	0.6669	0.0041	0.042*	
H9B	0.4426	0.6247	0.0137	0.042*	
C10	0.4279 (9)	0.6926 (2)	0.2083 (6)	0.0645 (18)	
H10A	0.3126	0.6775	0.1741	0.077*	
H10B	0.4458	0.7219	0.1464	0.077*	
C11	0.4376 (14)	0.7133 (3)	0.3593 (8)	0.099 (3)	
H11A	0.3489	0.7407	0.3593	0.118*	
H11B	0.4118	0.6842	0.4192	0.118*	
C12	0.7298 (13)	0.6951 (3)	0.4179 (7)	0.100 (3)	
H12A	0.7050	0.6656	0.4769	0.119*	
H12B	0.8438	0.7097	0.4592	0.119*	
C13	0.7348 (9)	0.6746 (2)	0.2692 (6)	0.0619 (16)	
H13A	0.7629	0.7038	0.2106	0.074*	
H13B	0.8256	0.6476	0.2735	0.074*	
C14	0.8215 (7)	0.5553 (2)	0.5089 (6)	0.0561 (14)	
H14A	0.8423	0.5183	0.4908	0.084*	
H14B	0.8673	0.5633	0.6064	0.084*	
H14C	0.8788	0.5773	0.4492	0.084*	
H3A	0.592 (8)	0.554 (2)	0.547 (4)	0.080*	

### Atomic displacement parameters (Å^2^)

**Table:**

	*U*^11^	*U*^22^	*U*^33^	*U*^12^	*U*^13^	*U*^23^
Zn1	0.0288 (3)	0.0448 (3)	0.0309 (3)	0.0064 (2)	0.0093 (2)	−0.0008 (2)
I1	0.02973 (19)	0.0787 (3)	0.0568 (2)	0.00815 (16)	0.01119 (16)	0.01217 (18)
Br1	0.0901 (5)	0.0545 (4)	0.0895 (5)	0.0392 (3)	0.0386 (4)	0.0100 (3)
O1	0.046 (2)	0.0353 (17)	0.0463 (19)	0.0053 (14)	0.0257 (17)	0.0043 (14)
O2	0.253 (9)	0.041 (3)	0.068 (3)	−0.004 (4)	0.044 (4)	−0.013 (2)
O3	0.0370 (18)	0.0466 (19)	0.0286 (16)	−0.0013 (14)	0.0093 (14)	0.0038 (14)
N1	0.0282 (19)	0.0265 (18)	0.0288 (18)	0.0003 (14)	0.0067 (15)	0.0010 (14)
N2	0.053 (3)	0.0282 (19)	0.037 (2)	0.0047 (17)	0.0179 (19)	0.0044 (15)
C1	0.032 (2)	0.030 (2)	0.027 (2)	0.0010 (18)	0.0060 (19)	−0.0024 (17)
C2	0.038 (3)	0.035 (2)	0.030 (2)	−0.0023 (19)	0.008 (2)	−0.0002 (18)
C3	0.066 (4)	0.036 (3)	0.044 (3)	0.004 (2)	0.027 (3)	0.007 (2)
C4	0.075 (4)	0.029 (2)	0.048 (3)	0.005 (2)	0.014 (3)	0.005 (2)
C5	0.046 (3)	0.038 (3)	0.040 (3)	0.012 (2)	0.010 (2)	−0.005 (2)
C6	0.037 (3)	0.037 (2)	0.037 (2)	0.004 (2)	0.013 (2)	−0.0018 (19)
C7	0.029 (2)	0.038 (2)	0.027 (2)	−0.0031 (19)	0.0084 (18)	−0.0034 (18)
C8	0.042 (3)	0.034 (2)	0.036 (2)	0.000 (2)	0.015 (2)	0.0049 (19)
C9	0.038 (3)	0.033 (2)	0.033 (2)	0.0041 (19)	0.009 (2)	0.0095 (18)
C10	0.107 (5)	0.039 (3)	0.057 (3)	0.030 (3)	0.041 (4)	0.017 (2)
C11	0.174 (10)	0.065 (5)	0.073 (5)	0.055 (5)	0.064 (6)	0.022 (4)
C12	0.196 (10)	0.046 (4)	0.051 (4)	−0.041 (5)	0.007 (5)	−0.009 (3)
C13	0.087 (5)	0.046 (3)	0.053 (3)	−0.026 (3)	0.013 (3)	−0.005 (2)
C14	0.040 (3)	0.075 (4)	0.050 (3)	−0.008 (3)	0.001 (3)	0.017 (3)

### Geometric parameters (Å, °)

**Table:**

Zn1—N1	2.014 (3)	C4—H4	0.9300
Zn1—O3	2.023 (3)	C5—C6	1.355 (6)
Zn1—O1	2.078 (3)	C6—H6	0.9300
Zn1—I1	2.5346 (9)	C7—H7	0.9300
Br1—C5	1.913 (4)	C8—C9	1.507 (6)
O1—C2	1.311 (5)	C8—H8A	0.9700
O2—C12	1.394 (10)	C8—H8B	0.9700
O2—C11	1.398 (11)	C9—H9A	0.9700
O3—C14	1.425 (6)	C9—H9B	0.9700
O3—H3A	0.84 (5)	C10—C11	1.528 (9)
N1—C7	1.285 (5)	C10—H10A	0.9700
N1—C8	1.478 (5)	C10—H10B	0.9700
N2—C10	1.472 (6)	C11—H11A	0.9700
N2—C13	1.478 (7)	C11—H11B	0.9700
N2—C9	1.481 (5)	C12—C13	1.525 (8)
C1—C6	1.411 (6)	C12—H12A	0.9700
C1—C2	1.414 (6)	C12—H12B	0.9700
C1—C7	1.442 (6)	C13—H13A	0.9700
C2—C3	1.405 (6)	C13—H13B	0.9700
C3—C4	1.375 (7)	C14—H14A	0.9600
C3—H3	0.9300	C14—H14B	0.9600
C4—C5	1.383 (7)	C14—H14C	0.9600
			
N1—Zn1—O3	114.78 (13)	C9—C8—H8A	109.4
N1—Zn1—O1	90.15 (12)	N1—C8—H8B	109.4
O3—Zn1—O1	90.42 (13)	C9—C8—H8B	109.4
N1—Zn1—I1	130.76 (10)	H8A—C8—H8B	108.0
O3—Zn1—I1	113.36 (9)	N2—C9—C8	112.2 (4)
O1—Zn1—I1	99.31 (9)	N2—C9—H9A	109.2
C2—O1—Zn1	126.0 (3)	C8—C9—H9A	109.2
C12—O2—C11	109.3 (5)	N2—C9—H9B	109.2
C14—O3—Zn1	118.2 (3)	C8—C9—H9B	109.2
C14—O3—H3A	109 (4)	H9A—C9—H9B	107.9
Zn1—O3—H3A	118 (4)	N2—C10—C11	110.1 (5)
C7—N1—C8	115.5 (3)	N2—C10—H10A	109.6
C7—N1—Zn1	124.4 (3)	C11—C10—H10A	109.6
C8—N1—Zn1	119.9 (3)	N2—C10—H10B	109.6
C10—N2—C13	107.9 (4)	C11—C10—H10B	109.6
C10—N2—C9	108.4 (4)	H10A—C10—H10B	108.2
C13—N2—C9	110.8 (4)	O2—C11—C10	112.5 (6)
C6—C1—C2	119.8 (4)	O2—C11—H11A	109.1
C6—C1—C7	115.6 (4)	C10—C11—H11A	109.1
C2—C1—C7	124.6 (4)	O2—C11—H11B	109.1
O1—C2—C3	120.1 (4)	C10—C11—H11B	109.1
O1—C2—C1	123.2 (4)	H11A—C11—H11B	107.8
C3—C2—C1	116.7 (4)	O2—C12—C13	111.4 (6)
C4—C3—C2	122.8 (4)	O2—C12—H12A	109.4
C4—C3—H3	118.6	C13—C12—H12A	109.4
C2—C3—H3	118.6	O2—C12—H12B	109.4
C3—C4—C5	118.9 (4)	C13—C12—H12B	109.4
C3—C4—H4	120.5	H12A—C12—H12B	108.0
C5—C4—H4	120.5	N2—C13—C12	110.2 (6)
C6—C5—C4	121.0 (4)	N2—C13—H13A	109.6
C6—C5—Br1	119.3 (4)	C12—C13—H13A	109.6
C4—C5—Br1	119.6 (4)	N2—C13—H13B	109.6
C5—C6—C1	120.7 (4)	C12—C13—H13B	109.6
C5—C6—H6	119.7	H13A—C13—H13B	108.1
C1—C6—H6	119.7	O3—C14—H14A	109.5
N1—C7—C1	128.3 (4)	O3—C14—H14B	109.5
N1—C7—H7	115.8	H14A—C14—H14B	109.5
C1—C7—H7	115.8	O3—C14—H14C	109.5
N1—C8—C9	111.1 (3)	H14A—C14—H14C	109.5
N1—C8—H8A	109.4	H14B—C14—H14C	109.5

### Hydrogen-bond geometry (Å, °)

**Table:**

*D*—H···*A*	*D*—H	H···*A*	*D*···*A*	*D*—H···*A*
O3—H3A···O1^i^	0.84 (5)	1.81 (5)	2.649 (4)	178 (7)

Symmetry codes: (i) −*x*+1, −*y*+1, −*z*+1.
